# Além do Volume de Contraste: Determinantes Relacionados ao Paciente e Considerações Metodológicas na Nefropatia Induzida por Contraste

**DOI:** 10.36660/abc.20260111

**Published:** 2026-06-16

**Authors:** Ömer Faruk Yilmaz, Yusuf Ziya Şener

**Affiliations:** 1 Ministry of Health Kaman State Hospital Department of Cardiology Kaman Kırşehir Turquia Ministry of Health Kaman State Hospital – Department of Cardiology, Kaman, Kırşehir – Turquia; 2 Ministry of Health Yavuzeli District State Hospital Department of Internal Medicine Yavuzeli Gaziantep Turquia Ministry of Health Yavuzeli District State Hospital – Department of Internal Medicine, Yavuzeli, Gaziantep – Turquia

**Keywords:** Nefropatias, Meios de Contraste, Intervenção Coronária Percutânea

**Prezado Editor,**

Lemos com grande interesse o artigo de Freitas et al., intitulado "*Impacto do Volume de Contraste Utilizado Após Procedimentos Coronários Percutâneos em Pacientes Predispostos à Nefropatia Induzida por Contraste*". Ao avaliar a interação entre o tipo e o volume de contraste por meio de uma análise post hoc, os autores abordaram uma questão clinicamente relevante em uma população de alto risco tratada com estratégias preventivas contemporâneas, oferecendo uma valiosa contribuição à literatura.^1^

No entanto, acreditamos que a interpretação dos resultados poderia ser aprimorada pela integração de considerações metodológicas e fisiopatológicas que vão além dos parâmetros relacionados ao contraste. O efeito protetor estatisticamente significativo do uso de estatinas contra a nefropatia induzida por contraste (NIC) é particularmente notável e corroborado por evidências randomizadas anteriores.^2,3^ Notavelmente, o estudo PRATO-ACS^4^ demonstrou que a administração precoce de altas doses de rosuvastatina reduziu significativamente a lesão renal aguda induzida por contraste por meio de efeitos pleiotrópicos anti-inflamatórios, antioxidantes e estabilizadores endoteliais, em grande parte independentes do volume de contraste. A coexistência de um efeito neutro do contraste com um benefício farmacológico significativo reforça a ideia de que a suscetibilidade à NIC pode ser impulsionada predominantemente pela vulnerabilidade vascular, inflamatória e relacionada à oxigenação do paciente, e não pelas propriedades físico-químicas dos agentes de contraste.^5^

Nesse contexto, a ausência de avaliação da hemoglobina representa uma limitação relevante. Murakami et al.^6^ identificaram a anemia como um preditor independente de NIC, particularmente em pacientes com reserva renal comprometida, por exacerbar a hipóxia medular renal e a suscetibilidade isquêmica após a exposição ao contraste. A falha em considerar esse componente de fornecimento de oxigênio pode, portanto, ter atenuado ou obscurecido potenciais associações entre os parâmetros de contraste e o risco de NIC. Essas observações estão em consonância com as perspectivas contemporâneas que enfatizam o papel central da inflamação sistêmica e do estresse metabólico na lesão renal associada ao contraste.

Da mesma forma, avaliar a terapia hipoglicemiante oral como uma categoria única e homogênea limita substancialmente a interpretabilidade. Evidências crescentes indicam que os inibidores do cotransportador sódio-glicose 2 (SGLT-2) exercem efeitos hemodinâmicos e metabólicos renais distintos, incluindo reduções no consumo tubular de oxigênio e melhorias no equilíbrio de oxigênio medular, o que pode se traduzir em menor suscetibilidade à NIC.^7,8^ A falta de estratificação por classe de medicamento antidiabético pode, portanto, ter obscurecido a heterogeneidade metabólica e microvascular clinicamente relevante. Além disso, a ausência de dados sobre o manejo perioperatório de agentes como a metformina introduz ainda mais incertezas, uma vez que a alteração no metabolismo do lactato em condições de hipóxia pode complicar a avaliação renal baseada na creatinina e refletir uma vulnerabilidade metabólica subjacente, em vez de toxicidade específica do medicamento.^9^

Outra consideração importante é que a estratificação post hoc pelo volume absoluto de contraste pode ter comprometido a comparabilidade basal e introduzido viés de indicação. Dados contemporâneos de intervenção coronária percutânea mostram consistentemente que o volume de contraste isoladamente reflete inadequadamente a carga nefrotóxica, a menos que seja contextualizado pela função renal basal.^10^ Nesse sentido, a maior TFGe basal entre os pacientes que receberam ≥150 mL de iodixanol provavelmente reflete uma alocação permissiva para aqueles considerados com maior reserva renal, enquanto a maior prevalência de cirurgia prévia de revascularização do miocárdio no mesmo subgrupo indica maior complexidade do procedimento, exigindo maior uso de contraste. Essa seleção bidirecional pode ter diluído substancialmente as verdadeiras associações entre os parâmetros de contraste e a NIC.^11^

Por fim, a predominância da população geriátrica impõe limitações fundamentais à avaliação renal baseada na creatinina. Grandes estudos populacionais têm demonstrado consistentemente que a redução da massa muscular esquelética relacionada à idade leva a níveis séricos basais de creatinina mais baixos, resultando em subestimação sistemática da disfunção renal real em idosos.^12^ Consequentemente, a dependência de definições baseadas na creatinina pode mascarar lesões renais subclínicas e subestimar a incidência de nefropatia induzida por contraste, particularmente em populações caracterizadas por fragilidade ou sarcopenia.^13^ Essa limitação é ainda agravada pelo declínio fisiológico da filtração glomerular relacionado à idade, que pode ser erroneamente classificado como função renal preservada quando se utiliza apenas a creatinina, atenuando assim a detecção de lesão renal associada ao contraste em pacientes idosos.^14^

Nesse contexto, embora o presente estudo forneça dados importantes sobre a seleção do contraste, futuras investigações prospectivas que incorporem estruturas compostas de avaliação de risco — como a TFGe basal, o escore de risco de Mehran, anemia e marcadores de fragilidade biológica, incluindo sarcopenia — podem permitir uma estratificação do risco de NIC mais coerente do ponto de vista fisiopatológico e clinicamente relevante.
