# Supplementary material for: Além do Volume de Contraste: Determinantes Relacionados ao Paciente e Considerações Metodológicas na Nefropatia Induzida por Contraste
Source: Arq Bras Cardiol. 2026 Jun 16;123(5):e20260111. [Article in Portuguese] doi: 10.36660/abc.20260111 (PMC13399492; doi:10.36660/abc.20260111)
Supplement: Translation

# Translation: Beyond Contrast Volume: Patient-Related Determinants and Methodological Considerations in Contrast-Induced Nephropathy

**DOI:** 10.36660/abc.20260111i

**Authors:** Ömer Faruk Yilmaz, Yusuf Ziya Şener

**Affiliations:** 1 Kaman Kırşehir Türkiye Ministry of Health Kaman State Hospital – Department of Cardiology, Kaman, Kırşehir – Türkiye; 2 Yavuzeli Gaziantep Türkiye Ministry of Health Yavuzeli District State Hospital – Department of Internal Medicine, Yavuzeli, Gaziantep – Türkiye

**Keywords:** Kidney Diseases, Contrast Media, Percutaneous Coronary Intervention

**Dear Editor,**

We read with great interest the article by Freitas et al. ^1^, entitled "*Impact of Contrast Volume Used After Percutaneous Coronary Procedures in Patients at Risk for Contrast-Induced Nephropathy*". By evaluating the interaction between contrast type and volume through a post hoc analysis, the authors addressed a clinically relevant question in a high-risk population managed with contemporary preventive strategies, providing a valuable contribution to the literature.^1^

Nevertheless, we believe that the interpretation of the findings could be strengthened by integrating methodological and pathophysiological considerations beyond contrast-related parameters alone. The statistically significant protective effect of statin use against contrast-induced nephropathy (CIN) is particularly noteworthy and supported by prior randomized evidence.^2,3^ Notably, the PRATO-ACS trial^4^ demonstrated that early high-dose rosuvastatin significantly reduced contrast-induced acute kidney injury through pleiotropic anti-inflammatory, antioxidant, and endothelial-stabilizing effects, largely independent of contrast volume. The coexistence of a neutral contrast effect with a meaningful pharmacological benefit supports the concept that CIN susceptibility may be driven predominantly by patient-related vascular, inflammatory, and oxygenation-related vulnerability rather than by the physicochemical properties of contrast agents.^5^

Within this framework, the absence of hemoglobin assessment represents a relevant limitation. Murakami et al.^6^ identified anemia as an independent predictor of CIN, particularly in patients with impaired renal reserve, by exacerbating renal medullary hypoxia and ischemic susceptibility following contrast exposure. Failure to account for this oxygen-delivery component may therefore have attenuated or obscured potential associations between contrast parameters and CIN risk. These observations align with contemporary perspectives emphasizing the central role of systemic inflammation and metabolic stress in contrast-associated renal injury.

Similarly, evaluating oral hypoglycemic therapy as a single, homogeneous category substantially limits interpretability. Growing evidence indicates that sodium–glucose cotransporter-2 (SGLT-2) inhibitors exert distinct renal hemodynamic and metabolic effects, including reductions in tubular oxygen consumption and improvements in medullary oxygen balance, which may translate into lower CIN susceptibility.^7,8^ Lack of stratification by antidiabetic drug class may thus have obscured clinically relevant metabolic and microvascular heterogeneity. In addition, the absence of data regarding peri-procedural management of agents such as metformin introduces further uncertainty, as altered lactate handling under hypoxic conditions may complicate creatinine-based renal assessment and reflect underlying metabolic vulnerability rather than drug-specific toxicit.^9^

Another important consideration is that post hoc stratification by absolute contrast volume may have compromised baseline comparability and introduced confounding by indication. Contemporary percutaneous coronary intervention data consistently show that contrast volume alone inadequately reflects nephrotoxic burden unless contextualized by baseline renal function.^10^ In this regard, the higher baseline eGFR among patients receiving ≥150 mL of iodixanol likely reflects permissive allocation to those perceived to have greater renal reserve, whereas the higher prevalence of prior coronary artery bypass grafting in the same subgroup indicates increased procedural complexity necessitating greater contrast use. This bidirectional selection may have substantially diluted true associations between contrast parameters and CIN.^11^

Finally, the predominance of a geriatric population introduces fundamental limitations to creatinine-based renal assessment. Large population-based studies have consistently demonstrated that age-related declines in skeletal muscle mass lead to lower baseline serum creatinine levels, resulting in systematic underestimation of true renal dysfunction in older adults.^12^ As a consequence, reliance on creatinine-based definitions may obscure subclinical kidney injury and underestimate the incidence of contrast-induced nephropathy, particularly in populations characterized by frailty or sarcopenia.^13^ This limitation is further compounded by the physiological age-related decline in glomerular filtration, which may be misclassified as preserved renal function when creatinine alone is used, thereby attenuating the detection of contrast-associated renal injury in elderly cohorts.^14^

In this context, while the present study provides important data regarding contrast selection, future prospective investigations incorporating composite risk assessment frameworks—such as baseline eGFR, the Mehran risk score, anemia, and markers of biological frailty, including sarcopenia—may allow for a more pathophysiologically coherent and clinically relevant stratification of CIN risk.
