# Supplementary material for: Além do Volume de Contraste: Determinantes Relacionados ao Paciente e Considerações Metodológicas na Nefropatia Induzida por Contraste
Source: Arq Bras Cardiol. 2026 Jun 16;123(5):e20260111. [Article in Portuguese] doi: 10.36660/abc.20260111 (PMC13399492; doi:10.36660/abc.20260111)
Supplement: Related texts

# Reply: Carta-resposta

**Authors:** Rafaela Andrade Penalva Freitas, José de Ribamar Costa

**Affiliations:** 1 Instituto Dante Pazzanese de Cardiologia São Paulo SP Brasil Instituto Dante Pazzanese de Cardiologia, São Paulo, SP – Brasil; 2 Hospital São Domingos São Luís MA Brasil Hospital São Domingos, São Luís, MA – Brasil

**Prezado Editor,**

Agradecemos sinceramente a leitura atenta e as considerações criteriosas acerca do nosso artigo "*Impacto do Volume de Contraste Utilizado Após Procedimentos Coronários Percutâneos em Pacientes Predispostos à Nefropatia Induzida por Contraste*", publicado nos Arquivos Brasileiros de Cardiologia em dezembro de 2025. Recebemos os comentários com grande apreço, pois enriquecem o debate científico e contribuem para o aprimoramento contínuo da investigação clínica.^1^

Gostaríamos de esclarecer alguns pontos relevantes que foram abordados na carta.

O estudo randomizado IDPC é o primeiro estudo randomizado com grande número de participantes a comparar os meios de contraste iodixanol e ioxaglato quanto à prevenção de nefropatia induzida por contraste (NIC) após intervenção coronária percutânea ou cateterismo diagnóstico. O principal achado dessa investigação é que não houve diferença significativa entre os meios de contraste iodixanol e ioxaglato quanto ao desenvolvimento de NIC em população de alto risco após procedimento diagnóstico ou terapêutico em laboratório de hemodinâmica de centro hospitalar terciário de cardiologia.^2^ Da mesma forma, na análise pós-hoc não houve associação entre o tipo de contraste e a ocorrência de NIC, mesmo entre pacientes expostos a maiores volumes dos dois meios de contraste.^3^

Em relação ao uso de estatinas, concordamos plenamente com a importância de seus efeitos pleiotrópicos na prevenção da nefropatia induzida por contraste (NIC), conforme evidenciado em estudos prévios. Em nossa coorte, 77% dos pacientes se encontravam em uso de estatina, refletindo a prática contemporânea de prevenção cardiovascular otimizada. Este dado não foi publicado neste artigo, mas encontrasse na integra na tese de doutorado (2021).^4^ Esse alto percentual pode possivelmente contribuir para a atenuação de diferenças adicionais relacionadas exclusivamente aos parâmetros do contraste.

Quanto à anemia, reconhecemos seu papel fisiopatológico na hipóxia medular renal e na vulnerabilidade à lesão renal aguda. Ressaltamos, contudo, que a maioria dos nossos pacientes apresentava níveis de hemoglobina dentro da normalidade. Além disso, esse parâmetro foi contemplado indiretamente por meio da incorporação do escore de Mehran, que inclui anemia como variável de risco, contribuindo para uma estratificação clínica já validada. Apresento a Tabela 1 sobre Escore Mehran e probabilidade de sobrevida de acordo com o tipo de contraste.^4^

**Tabela 1:** Escore Mehran e probabilidade de sobrevida de acordo com tipo de contraste

Escore Mehran							
Variável			≤ 5	6 – 10	11 – 15	> 15	p-valor
Tipo	Total	n (%)	386 (20,6)	885 (47,2)	523 (27,9)	81 (4,3)	
	Ioxaglato	n (%)	195 (50,5)	432 (48,8)	264 (50,5)	31 (38,3)	
	Iodixanol	n (%)	191 (49,5)	453 (51,2)	259 (49,5)	50 (61,7)	0,209
Probabilidade Sobrevida Óbito		(%)	97,7%	97,1%	96,9%	92,6%	0,081 *
Probabilidade Sobrevida Combinado		(%)	97,2%	96,8%	95,4%	87,8%	0,006 *

p-valor obtido pelo teste U de Mann-Whitney.

* p-valor obtido pelo teste de log-rank. Tabela extraída da tese de doutorado.^4^

No que se refere aos inibidores de SGLT-2, concordamos plenamente quanto aos seus relevantes efeitos hemodinâmicos e metabólicos renais. Entretanto, no período em que os procedimentos foram realizados no serviço de hemodinâmica, essa classe terapêutica ainda não estava disponível no Brasil, motivo pelo qual nenhum dos pacientes fazia uso dessas medicações. Assim, não houve potencial impacto dessa variável na análise.

Sobre os dados do manejo periprocedimento de agentes como a metformina, vale ressaltar que é rotina do serviço suspender a medicação 48 horas antes do procedimento a fim de diminuir os riscos e complicações relacionados à acidose lática.

Em relação ao protocolo de hidratação, todos os pacientes incluídos no estudo colheram a creatinina no período de 48 a 96h após a realização de cateterismo diagnóstico ou terapêutico.

Acrescenta-se que os pacientes com taxa de filtração ≤ 45mL/min foram internados para hidratação endovenosa e aqueles que já haviam apresentado quadro prévio de NIC, mesmo com clearence > 45 mL/min.

Aqueles pacientes com clearance ≤ 45mL/min receberam hidratação endovenosa com solução salina comum (0,9%), nas doses de 0,5 a 1,0 mL/kg/min de acordo com a presença ou não de disfunção do ventrículo esquerdo, conhecida previamente através do ecocardiograma transtorácico, por no mínimo 12 horas antes do procedimento, a qual foi mantida durante a realização do exame e 12 horas após seu término.

Pacientes com clearance de creatinina entre 45 e 60 mL/min também foram hidratados por via endovenosa pré-procedimento, por 4 horas antes do ínicio do procedimento, durante o mesmo e por no mínimo 4 horas após.

Os pacientes com clearance de creatinina ≤ 45mL/min permaneceram internados até o dia seguinte quando foram realizadas as dosagens de ureia, creatinina, sódio e potássio antes da alta hospitalar para verificar se houve alteração adicional nos parâmetros de função renal. Se houvesse elevação dos mesmos, o paciente permanecia internado para hidratação, avaliação clínica e observação dos exames até a melhora parcial ou total do quadro. Aqueles que não apresentaram alterações na creatinina receberam alta hospitalar e foram orientados a colher a creatinina entre 48-96 horas após o procedimento conforme o protocolo.

Aqueles com clearance > 45 até 60 mL/min recebiam alta no mesmo dia, com orientação e pedido laboratorial para colher a creatinina entre 48-96h após a realização do procedimento com contraste.

Quanto à população estudada, destacamos que o predomínio de pacientes idosos reflete a realidade cotidiana do nosso serviço de hemodinâmica com elevado número de procedimentos por ano. Sabemos que muitos desses pacientes são sub-representados ou mesmo excluídos de grandes ensaios clínicos randomizados. Nosso objetivo foi justamente oferecer uma análise pragmática, centrada na prática clínica real, buscando elucidar uma questão recorrente do dia a dia assistencial: o real peso do volume absoluto de contraste em um cenário de prevenção contemporânea e em uma população de elevada complexidade clínica.

Concordamos que modelos prospectivos futuros incorporando variáveis adicionais — como marcadores mais sensíveis de função renal, fragilidade biológica e estratificações metabólicas mais refinadas — poderão aprofundar ainda mais a compreensão da vulnerabilidade individual à NIC. No entanto, acreditamos que nossos achados contribuem ao demonstrar que, em um contexto de prevenção otimizada e avaliação de risco consolidada, o volume absoluto de contraste pode não ser o determinante isolado predominante do desfecho renal.

Reiteramos nosso agradecimento pelas valiosas observações e pelo estímulo ao debate científico construtivo.

## References

[1] Freitas RAP, Tanajura LFL, Sousa AGMR, Feres F, Costa JR (2025). Impact of Contrast Volume Used after Percutaneous Coronary Procedures in Patients at Risk for Contrast-Induced Nephropathy. Arq Bras Cardiol.

[2] Freitas RAP, Tanajura LF, Mehran R, Chamié D, Chaves A, Centemero M (2023). Ioxaglate versus IoDixanol for the Prevention of Contrast-Induced Nephropathy: The IDPC Trial. J Invasive Cardiol.

[3] Freitas RAP, Tanajura LFL, Sousa AGMR, Feres F, Costa JR (2025). Impact of Contrast Volume Used after Percutaneous Coronary Procedures in Patients at Risk for Contrast-Induced Nephropathy. Arq Bras Cardiol.

[4] Freitas RAP (2021). Ioxaglato versus Iodixanol na Prevenção de Nefropatia Induzida por Contraste - Estudo Randomizado IDPC [Thesis].

# Reply

**Authors:** Rafaela Andrade Penalva Freitas, José de Ribamar Costa

**Affiliations:** 1 São Paulo SP Brazil Instituto Dante Pazzanese de Cardiologia, São Paulo, SP – Brazil; 2 São Luís MA Brazil Hospital São Domingos, São Luís, MA – Brazil

**Dear Editor,**

We sincerely appreciate your careful reading and thoughtful consideration of our article "Impact of Contrast Volume Used After Percutaneous Coronary Procedures in Patients at Risk for Contrast-Induced Nephropathy," published in the *Arquivos Brasileiros de Cardiologia* in December 2025. We greatly value your comments, as they enrich the scientific debate and contribute to the continuous improvement of clinical research.^1^

We would like to clarify some relevant points that were addressed in the letter.

The randomized IDPC study is the first large-scale randomized study to compare the contrast agents iodixanol and ioxaglate in preventing contrast-induced nephropathy (CIN) after percutaneous coronary intervention or diagnostic catheterization. The main finding of this investigation is that there was no significant difference between the contrast agents iodixanol and ioxaglate in the development of CIN in a high-risk population after diagnostic or therapeutic procedures in a hemodynamics laboratory of a tertiary cardiology hospital center.^2^ Similarly, in the post-hoc analysis, there was no association between the type of contrast agent and the occurrence of CIN, even among patients exposed to higher volumes of both contrast agents.^3^

Regarding the use of statins, we fully agree with the importance of their pleiotropic effects in preventing contrast-induced nephropathy (CIN), as evidenced in previous studies. In our cohort, 77% of patients were using statins, reflecting the contemporary practice of optimized cardiovascular prevention. This data was not published in this article, but can be found in full in the doctoral thesis (2021).^4^ This high percentage may possibly contribute to the attenuation of additional differences related exclusively to contrast parameters.

Regarding anemia, we recognize its pathophysiological role in renal medullary hypoxia and vulnerability to acute kidney injury. However, we emphasize that most of our patients had hemoglobin levels within the normal range. Furthermore, this parameter was indirectly addressed through the incorporation of the Mehran score, which includes anemia as a risk variable, contributing to a validated clinical stratification. The Table 1 below shows the Mehran score and probability of survival according to the type of contrast agent.^4^

**Table 1:** Mehran score and probability of survival according to type of contrast agent

Mehran Score							
Variable			≤ 5	6 – 10	11 – 15	> 15	p-value
Type	Total	n (%)	386 (20.6)	885 (47.2)	523 (27.9)	81 (4,3)	
	Ioxaglate	n (%)	195 (50.5)	432 (48.8)	264 (50.5)	31 (38.3)	
	Iodixanol	n (%)	191 (49.5)	453 (51.2)	259 (49.5)	50 (61.7)	0.209
Probability of Survival Death		(%)	97.7%	97.1%	96.9%	92.6%	0.081 *
Probability of Survival Combined		(%)	97.2%	96.8%	95.4%	87.8%	0.006 *

p-value obtained by the Mann-Whitney U test.

* p-value obtained from the log-rank test. Table extracted from the doctoral thesis.^4^

Regarding SGLT-2 inhibitors, we fully agree on their relevant hemodynamic and renal metabolic effects. However, during the period in which the procedures were performed in the hemodynamics service, this therapeutic class was not yet available in Brazil, which is why none of the patients were using these medications. Thus, there was no potential impact of this variable on the analysis.

Regarding data on the periprocedural management of agents such as metformin, it is worth noting that it is routine for the service to suspend the medication 48 hours before the procedure in order to reduce the risks and complications related to lactic acidosis.

Regarding the hydration protocol, all patients included in the study had their creatinine levels measured 48 to 96 hours after undergoing diagnostic or therapeutic catheterization.

It should be added that patients with a filtration rate ≤ 45 mL/min were admitted for intravenous hydration, as were those who had previously presented with CIN, even with a clearance > 45 mL/min.

Those patients with clearance ≤ 45 mL/min received intravenous hydration with ordinary saline solution (0.9%), at doses of 0.5 to 1.0 mL/kg/min according to the presence or absence of left ventricular dysfunction, previously known through transthoracic echocardiography, for at least 12 hours before the procedure, which was maintained during the examination and for 12 hours after its completion.

Patients with creatinine clearance between 45 and 60 mL/min were also hydrated intravenously pre-procedure, for 4 hours before the start of the procedure, during the procedure, and for at least 4 hours afterward.

Patients with creatinine clearance ≤ 45 mL/min remained hospitalized until the following day, when urea, creatinine, sodium, and potassium levels were measured before hospital discharge to check for any further changes in renal function parameters. If these parameters were elevated, the patient remained hospitalized for hydration, clinical evaluation, and monitoring of tests until partial or complete improvement. Those who did not show changes in creatinine were discharged and instructed to have their creatinine levels measured between 48 and 96 hours after the procedure, according to protocol.

Those with clearance > 45 up to 60 mL/min were discharged on the same day, with instructions and a laboratory request to collect creatinine between 48-96 hours after the contrast-enhanced procedure.

Regarding the study population, we highlight that the predominance of elderly patients reflects the daily reality of our hemodynamics service, with a high number of procedures per year. We know that many of these patients are underrepresented or even excluded from large randomized clinical trials. Our objective was precisely to offer a pragmatic analysis, centered on real clinical practice, seeking to elucidate a recurring issue in daily care: the real weight of the absolute volume of contrast in a contemporary prevention scenario and in a population of high clinical complexity.

We agree that future prospective models incorporating additional variables—such as more sensitive markers of renal function, biological frailty, and more refined metabolic stratifications—could further deepen the understanding of individual vulnerability to CIN. However, we believe that our findings contribute by demonstrating that, in a context of optimized prevention and consolidated risk assessment, the absolute volume of contrast may not be the predominant single determinant of renal outcome.

We reiterate our gratitude for the valuable observations and for encouraging constructive scientific debate.
